# Machine Learning Unveils Dietary Antioxidants as Influential Factors for Diabetes‐Cancer Comorbidity: Insights From National Health and Nutrition Examination Survey

**DOI:** 10.1002/fsn3.71828

**Published:** 2026-04-27

**Authors:** Ming Xu, Ru Li, Shuqing Jin, Qinhao Liu, Hongxia Wei, Zhe Li, Yuchen Sun, Yi Zhang, Yunfeng Liu

**Affiliations:** ^1^ Department of Endocrinology First Hospital of Shanxi Medical University Taiyuan China; ^2^ First Clinical Medical College Shanxi Medical University Taiyuan China; ^3^ Department of Pharmacology Shanxi Medical University Taiyuan China; ^4^ Shanxi Key Laboratory of Metabolic‐Immune Homeostasis and Drug Innovation Taiyuan China; ^5^ Clinical Research Center for Metabolic Diseases of Shanxi Medical University Taiyuan China

**Keywords:** cancer, machine learning, diabetes mellitus type 2, dietary antioxidants, National Health and Nutrition Examination Survey

## Abstract

This study aims to assess the predictive value of dietary antioxidants in diabetes‐cancer comorbidity using interpretable machine learning (ML) models and to identify key clinical factors. Data were sourced from the National Health and Nutrition Examination Survey (NHANES) 2007–2010 and 2017–2018 cycles, including 44 dietary antioxidants, as well as demographic, lifestyle, and health‐related features. 8 ML models (Random Forest, light Gradient Boosting Machines [LightGBM], Logistic Regression, Decision Tree, Multilayer Perceptron, Naïve Bayes, Kernel k‐Nearest Neighbors, and Support Vector Machine with Radial Basis Function) were trained, with preprocessing steps for multicollinearity, class imbalance (SMOTE), and data normalization. Model performance was evaluated using AUC, accuracy, Brier scores, and calibration plots. SHapley Additive exPlanations (SHAP) values were applied to interpret feature importance. Data from 8644 participants were analyzed, including 272 individuals with confirmed diabetes‐cancer comorbidity. After removing collinear features, the ML model included 30 dietary antioxidant features and 10 baseline features. The Random Forest model achieved optimal performance (AUC = 0.996, accuracy = 0.978, brier score = 0.0241), followed by LightGBM (AUC = 0.993). SHAP analysis revealed that while advanced age, cardiovascular disease, and hypertension were the primary drivers of comorbidity probability, dietary antioxidants are also influential factors. Specifically, polyphenols (daidzein, malvidin, pelargonidin, cyanidin) and essential minerals (magnesium) emerged as the most influential nutritional features. The high accuracy of the Random Forest and LightGBM models underscores their clinical utility in risk stratification for diabetes‐cancer comorbidity. While advancing age and cardiometabolic dysfunction primarily drives the probability of diabetes‐cancer comorbidity. This study establishes dietary antioxidants, particularly polyphenols such as daidzein and malvidin, as predictive factors for diabetes‐cancer comorbidity.

## Graphical abstract

This study employs an interpretable machine learning framework to predict diabetes‐cancer comorbidity, with the Random Forest and LightGBM models demonstrating exceptional performance. SHAP value analysis reveals that while advanced age and cardiometabolic dysfunctions serve as primary risk drivers, dietary antioxidants (particularly daidzein and malvidin) are important predictive factors. These findings provide novel data‐driven insights for personalized nutritional interventions.

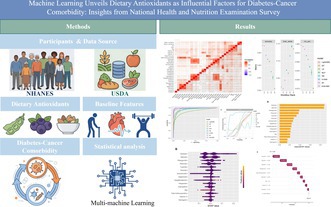

## 1 Introduction

Cancer and diabetes, two of the most pressing global public health challenges, are increasingly recognized for their interconnectedness and comorbidity (Wang et al. 2020). According to the World Health Organization, approximately 9.6 million deaths worldwide in 2018 were attributed to cancer, representing one‐sixth of all global deaths (de Martel et al. 2020). By 2021, the number of individuals living with diabetes had surged to 529 million, with prevalence rates continuing to climb (GBD 2021 Diabetes Collaborators 2023). Type 2 diabetes mellitus (T2DM), which constitutes the majority of diabetes cases, has been strongly linked to several cancers, including liver, pancreatic, and endometrial cancers (Buysschaert and Sadikot 2013; Giovannucci et al. 2010). A comprehensive meta‐analysis of 32 million individuals demonstrated that T2DM significantly elevates the risk of developing these cancers (Ling et al. 2020). Importantly, diabetes not only increases cancer incidence but is also associated with poorer treatment outcomes, higher complication rates, and reduced survival, creating a bidirectional and detrimental cycle (Ling et al. 2022; Mrzljak et al. 2022).

Pathophysiological studies have identified oxidative stress as a key mechanism linking these two diseases (Chen et al. 2022; Schwartz et al. 2019). Chronic hyperglycemia drives the overproduction of reactive oxygen species (ROS) through mitochondrial electron transport chain dysfunction, leading to oxidative DNA damage (Giacco and Brownlee 2010). Accumulating evidence suggests that other factors, such as dysregulation of the insulin/insulin‐like growth factor (IGF) axis, persistent hyperglycemia, inflammatory cytokines, and sex hormones, may also play pivotal roles in cancer development (Vigneri et al. 2009). Conversely, cancer treatments themselves can exacerbate metabolic dysregulation in diabetes. For instance, cancer survivors exhibit a higher incidence of diabetes, likely due to targeted therapies disrupting insulin receptor signaling pathways (Onitilo et al. 2013; Stava et al. 2007). While antidiabetic agents like metformin have demonstrated potential anticancer properties, many anticancer therapies may inadvertently worsen diabetes progression, making the clinical management of patients with diabetes and cancer comorbidities more challenging (Vigneri et al. 2009).

Dietary interventions, as modifiable and accessible strategies for disease prevention, offer promising avenues for mitigating these risks (Brown et al. 2015). Polyphenolic compounds, characterized by their unique aromatic ring structures and hydroxyl groups, exhibit dual antioxidant and anti‐inflammatory properties (Salisbury and Bronas 2015). These compounds not only neutralize ROS directly but also modulate the Nrf2/ARE pathway to mitigate oxidative stress and inhibit the NF‐κB signaling cascade to reduce inflammation (Ali et al. 2016; Braidy et al. 2019; Nimse and Pal 2015; Ribas et al. 2008; Tena et al. 2020).

Unlike traditional statistical approaches, machine learning (ML) techniques provide unparalleled flexibility, free from the constraints of prior assumptions. ML excels at analyzing large, complex datasets, uncovering hidden relationships among diverse health‐related factors, and offers innovative tools to model the intricate interplay between diet and diseases (Morgenstern et al. 2021; Rajula et al. 2020). While previous research has successfully applied ML to predict the independent risk of cancer or T2DM, the application of ML to predict the comorbidity of diabetes and cancer remains largely unexplored (Xue et al. 2020; Birk et al. 2021; Park et al. 2024; Abdul Rahman et al. 2023; Qarmiche et al. 2023). This study establishes its scientific novelty by employing an interpretable ML framework specifically targeting this comorbidity. By employing multidimensional data from the National Health and Nutrition Examination Survey (NHANES) database, we systematically investigated the complex relationship between demographic characteristics, clinical baseline features, 44 dietary antioxidants and diabetes‐cancer comorbidity. Crucially, we developed a robust performance comparison framework utilizing advanced algorithms, including Light Gradient Boosting Machines (lightGBM) and Random Forests. To overcome the traditional black‐box limitation inherent to complex algorithmic models, we integrated the SHapley Additive exPlanations (SHAP) algorithm, which allows for individualized quantification of feature importance. Consequently, this approach offers novel translational insights for therapeutic and preventive nutritional interventions.

## 2 Participants and Methods

### 2.1 Participants

**FIGURE 1:**
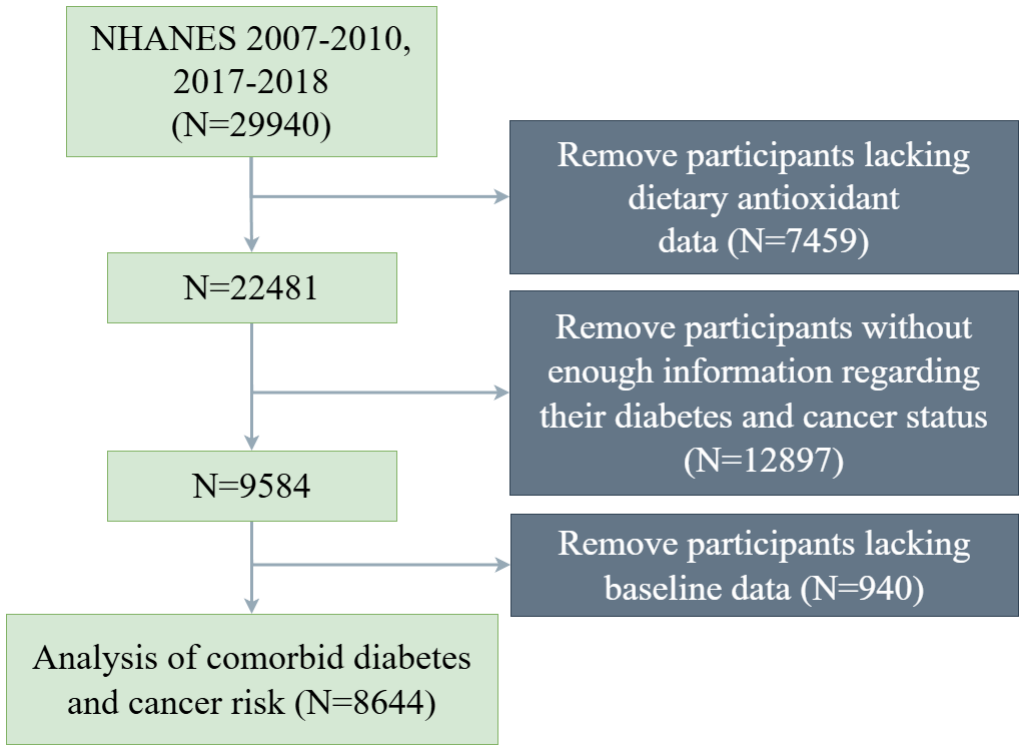
Participants screening flow chart.

The NHANES, conducted by the United States National Center for Health Statistics, utilizes a complex, stratified, multistage probability sampling design to gather demographic, socioeconomic, dietary, and health‐related data representative of the civilian, noninstitutionalized population (https://wwwn.cdc.gov/nchs/nhanes/tutorials/sampledesign.aspx). Participants evaluated in the 2007–2010 and 2017–2018 survey cycles were considered as the initial candidate pool for this study (*N* = 29,940). To ensure data integrity and methodological reproducibility, a stepwise exclusion protocol was applied to derive the final cohort. First, individuals lacking complete data for their 24‐h dietary recall interviews were excluded from the analysis (*N* = 7459). Second, individuals without enough diagnostic survey data or laboratory values regarding their diabetes or cancer status were excluded to ensure outcome classification (*N* = 12,897). Finally, participants with missing data across essential baseline clinical and demographic covariates were excluded to facilitate a complete case analysis framework for the machine learning algorithms (*N* = 940). Following this selection process, detailed comprehensively in Figure 1, the final participants were determined (*N* = 8644).

### 2.2 Dietary Antioxidant Intake

The intake data for 44 dietary antioxidants, including vitamins, minerals, and polyphenolic compounds, were extracted from NHANES. Dietary intake was assessed using the United States Department of Agriculture (USDA)'s Automated Multiple‐Pass Method, a highly validated, computerized, five‐step interview procedure specifically designed to minimize respondent recall bias and significantly improve the accuracy of self‐reported nutritional data (Stote et al. 2011). Survey participants underwent two detailed 24‐h dietary recall interviews. The initial recall was conducted face‐to‐face by trained interviewers within a specialized Mobile Examination Center, and the subsequent recall was administered via a structured telephone interview 3–10 days later.

To establish continuous dietary features for the machine learning algorithms, the unweighted mean daily intake of the selected dietary antioxidants was calculated for participants who successfully completed both 24‐h recalls. Because the second recall is typically conducted by phone 3–10 days after the first, taking the average of these two nonconsecutive days allows for the partial minimization of measurement errors associated with day‐to‐day variation in individual diets. Additionally, because individual dietary consumption patterns naturally fluctuate across time, seasonal variations were inherently mitigated by the continuous, year‐round operational design of the survey, which ensures all seasons are proportionally represented.

### 2.3 Diagnosis of Diabetes and Cancer

Participants are diagnosed with DM if they meet any of the following criteria: fasting plasma glucose (FPG) ≥ 126 mg/dL; 2‐h serum glucose ≥ 200 mg/dL following a 75 g glucose load in an oral glucose tolerance test (OGTT); HbA1c > 6.5%; self‐reported DM or the use of antihyperglycemic medications, as indicated in the NHANES questionnaire. Similarly, a cancer diagnosis was confirmed if participants reported being informed by a physician or other healthcare professional that they have cancer or specified a particular type of cancer in the medical conditions questionnaire.

While the diagnosis of diabetes was supported by highly objective physiological biomarker data, the classification of cancer status relied on self‐reported survey data. Although self‐reporting inherently introduces a potential vulnerability to recall bias and misclassification, extensive epidemiological validation studies have consistently demonstrated that self‐reported incident cancer diagnoses possess a high degree of validity in large, population‐based survey cohorts. For instance, comprehensive validation studies comparing self‐reported cancer histories directly to gold‐standard Medicare diagnostic claims and comprehensive state cancer registries have demonstrated an overall diagnostic sensitivity ranging from 73.2% to 93.0% and a highly robust specificity consistently exceeding 96.0% (Mullins et al. 2022). The consistently high specificity ensures that the non‐cancer control group within this study remains largely free of misclassified cancer cases.

### 2.4 Collection of Baseline Features

Baseline characteristics were collected based on three aspects including demographic, lifestyle, and health condition. These included age, gender (male or female), race/ethnicity (Mexican American, other Hispanic, non‐Hispanic white, non‐Hispanic black, and other categories), family poverty‐to‐income ratio (PIR), smoking status (yes or no), engagement in moderate to vigorous physical activity (yes or no), as well as the presence of hyperlipidemia, hypertension, and CVD. Data on age, gender, race/ethnicity, and family poverty‐to‐income ratio were retrieved from the Demographic Data module in NHANES, while information on smoking and physical activity was sourced from the Questionnaire Data module. Individuals who reported smoking fewer than 100 cigarettes over their lifetime were categorized as non‐smokers, while others were classified as smokers based on their response to the question “smoked at least 100 cigarettes in life?” The diagnoses of dyslipidemia and hypertension were established using both measurements and self‐reported data from the Questionnaire Data module. Specifically, hyperlipidemia was defined by high‐density lipoprotein cholesterol levels below 1.0 mmol/L in men, below 1.3 mmol/L in women, or triglycerides at or above 1.8 mmol/L for all participants. Hypertension was identified as having a systolic blood pressure ≥ 140 mmHg and/or diastolic blood pressure ≥ 90 mmHg on mean value of three occasions, or if participants answered “yes” to questions about taking prescribed medication for high blood pressure or being previously diagnosed with hypertension. CVD diagnosis was confirmed if participants reported being informed by a physician or other healthcare professional that they had congestive heart failure, coronary heart disease, angina, myocardial infarction, or stroke.

### 2.5 Pre‐Processing of Machine Learning Features

The dataset for this study initially consisted of 55 features, comprising 47 continuous variables and 8 categorical ones. To reduce multicollinearity among dietary antioxidant features, correlation coefficients were calculated, and features with coefficients exceeding 0.9 were removed. To address class imbalance in the dataset, we applied the Synthetic Minority Over‐sampling Technique (SMOTE) to ensure balanced representation of classes before model training. Specifically, for each minority class instance, SMOTE identifies its K‐nearest neighbors and creates new data points through linear interpolation along the line segments connecting the original instance to its selected neighbors. Finally, we standardized all features using the *z*‐score normalization to prevent variables with larger scales from dominating the model.

### 2.6 Statistical Analysis

The characteristics of participants with and without comorbidity disease were described using survey‐weighted statistical models. Continuous variables were presented as mean ± standard error, while categorical variables were expressed as frequencies and percentages. Characteristics were compared using the weighted *χ* ^2^ test for categorical variables, ANOVA for normally distributed continuous variables, and the Kruskal–Wallis H test for skewed distributions.

Discriminative models, including Light Gradient Boosting Machine (LightGBM), Decision Tree (RPART), Logistic Regression (LR), Multilayer Perceptron (MLP), Naïve Bayes (NB), Kernel k‐Nearest Neighbors (K‐KNN), Random Forest (RF), and Support Vector Machine with Radial Basis Function (SVM‐RBF), were developed using the tidymodels framework in R. LightGBM is particularly suitable for large‐scale datasets due to its computational efficiency, while maintaining high prediction accuracy (Yang, Chen, et al. 2023). The RPART model effectively captures nonlinear relationships and feature interactions, making it ideal for handling complex variable relationships in data (Strobl et al. 2009). LR remains a fundamental choice for risk prediction because of its simplicity and interpretability, while MLP excels at identifying intricate patterns in data (Bishop 1991; Boateng and Abaye 2019). NB is computationally efficient, especially for large‐scale datasets (Langarizadeh and Moghbeli 2016). The K‐KNN model classifies samples based on their similarities, making it well‐suited for data with uneven sample distributions (Mullick et al. 2018). RF is capable of modeling non‐linear relationships and complex feature interactions, whereas SVM is effective for high‐dimensional data (Son et al. 2010; Unnikrishnan et al. 2016; Xin and Ren 2022). These models have also been successfully employed in analyzing NHANES data in previous studies, further demonstrating their applicability (Lundberg and Lee 2017; Tan et al. 2024; Vollmer et al. 2022).

To evaluate model performance, we conducted comprehensive discrimination and calibration analyses. Receiver operating characteristic (ROC) curves were generated to calculate the area under the curve (AUC), with the AUC‐ROC metric quantifying the model's ability to differentiate between outcome classes. Calibration was assessed through brier scores and calibration plots, where the brier score measures the mean squared deviation between predicted probabilities and actual outcomes (lower values indicating superior calibration). Classification accuracy, defined as the proportion of correctly predicted instances relative to the total sample, provided additional performance validation.

To enhance model interpretability, we employed SHAP values for global feature importance assessment in machine learning models demonstrating optimal predictive performance. As the frontier in explainable artificial intelligence, SHAP employs game‐theoretic principles to aggregate local feature contributions, thereby elucidating global model behavior through additive feature attribution. This method has demonstrated superiority over conventional global approximation approaches in recent methodological comparisons (Lundberg and Lee 2017). The algorithm provides dual capability: (1) comprehensive feature importance quantification at the population level, and (2) granular insights into feature effects at individual prediction levels.

All analyses were conducted using R statistical software (version 4.4.1) with the following specialized packages: dplyr for data manipulation, themis for class imbalance correction, baguette for ensemble modeling, discrim for discriminant analysis, probably for probability calibration, treeshap for SHAP value computation, and shapviz for interactive visualization of SHAP outputs. Statistical tests were two‐sided and a *p*‐value < 0.05 was considered statistically significant.

## 3 Results

### 3.1 Characteristics of the Features

A total of 8644 participants were included in this analysis, and 272 of them were identified as having comorbidity of diabetes and cancer. Compared to participants without comorbidity, those with comorbid conditions had significantly lower intakes of glycitein (0.18 [1.00] vs. ±0.06 [0.42]) and kaempferol (4.83 [6.21] vs. 4.05 [5.83]), significantly higher intakes of vitamin A (650.33 [489.57] vs. 750.96 [648.35]) and beta‐carotene (2322.05 [3213.76] vs. 2474.03 [2630.72]). There were significant differences between the two groups in terms of age, race, physical activity, hypertension condition, hyperlipidemia condition and CVD condition (Table 1).

**TABLE 1:** Characteristics of enrolled participants.

Characteristic	*N* ^a^	Overall *N* = 121,603,555^b^	Non‐comorbid diabetes and cancer *N* = 118,289,553^b^	Comorbid diabetes and cancer *N* = 3,314,002^b^	*p*‐Value^c^
Gender	8644				0.343
Male		4127 (48%)	3982 (48%)	145 (52%)	
Female		4517 (52%)	4390 (52%)	127 (48%)	
Age	8644	47.93 ± (16.93)	47.33 ± (16.68)	69.37 ± (10.43)	< 0.001
Race	8644				< 0.001
Mexican American		1286 (14.8%)	1261 (15.1%)	25 (9.1%)	
Other Hispanic		799 (9.2%)	781 (9.3%)	18 (6.6%)	
Non‐Hispanic White		3957 (45.7%)	3775 (45.1%)	182 (66.9%)	
Non‐Hispanic Black		1742 (20.1%)	1708 (20.4%)	34 (12.5%)	
Other race		860 (9.9%)	847 (10.1%)	13 (4.8%)	
Education	8644				0.591
Less than 9th grade		769 (4.0%)	738 (3.9%)	31 (6.1%)	
9–11th grade		1174 (9.6%)	1136 (9.6%)	38 (9.4%)	
High School Grad/GED or Equivalent		2020 (25%)	1960 (25%)	60 (25%)	
Some College or AA degree		2670 (31%)	2582 (31%)	88 (33%)	
College Graduate or above		2011 (31%)	1956 (31%)	55 (27%)	
Family PIR	8644	3.08 ± (1.63)	3.08 ± (1.63)	3.23 ± (1.58)	0.256
Smoke	8644				0.078
Yes		3912 (44%)	3769 (43%)	143 (52%)	
No		4732 (56%)	4603 (57%)	129 (48%)	
Moderate to vigorous physical activity	8644				< 0.001
No		4615 (46%)	4416 (45%)	199 (66%)	
Yes		4029 (54%)	3956 (55%)	73 (34%)	
Hypertension	8644				< 0.001
No		4833 (62%)	4786 (63%)	47 (23%)	
Yes		3811 (38%)	3586 (37%)	225 (77%)	
Hyperlipidemia	8644				< 0.001
No		5683 (67%)	5538 (68%)	145 (47%)	
Yes		2961 (33%)	2834 (32%)	127 (53%)	
CVD	8644				< 0.001
No		7624 (91%)	7450 (92%)	174 (66%)	
Yes		1020 (9.0%)	922 (8.3%)	98 (34%)	
Vitamin A	8644	653.07 ± (494.80)	650.33 ± (489.57)	750.96 ± (648.35)	0.004
Vitamin C	8644	80.63 ± (73.78)	80.76 ± (74.16)	75.96 ± (58.53)	0.923
Vitamin E	8644	8.59 ± (5.52)	8.59 ± (5.54)	8.39 ± (4.71)	0.852
Mg	8644	300.97 ± (130.00)	301.30 ± (130.49)	289.00 ± (110.60)	0.395
Zinc	8644	11.49 ± (6.83)	11.49 ± (6.86)	11.53 ± (5.55)	0.584
Se	8644	112.86 ± (51.72)	112.93 ± (51.73)	110.15 ± (51.40)	0.473
Alpha‐carotene	8644	403.44 ± (887.28)	403.50 ± (892.09)	401.60 ± (695.03)	0.120
Beta‐carotene	8644	2326.19 ± (3199.26)	2322.05 ± (3213.76)	2474.03 ± (2630.72)	0.003
Daidzein	8644	0.84 ± (4.31)	0.86 ± (4.36)	0.33 ± (1.81)	0.636
Genistein	8644	1.20 ± (6.11)	1.22 ± (6.18)	0.43 ± (2.41)	0.132
Glycitein	8644	0.18 ± (0.99)	0.18 ± (1.00)	0.06 ± (0.42)	< 0.001
Cyanidin	8644	2.68 ± (9.63)	2.68 ± (9.71)	2.64 ± (5.99)	0.294
Petunidin	8644	1.38 ± (5.14)	1.37 ± (5.13)	1.75 ± (5.22)	0.602
Delphinidin	8644	1.95 ± (7.21)	1.95 ± (7.24)	2.07 ± (5.88)	0.544
Malvidin	8644	5.31 ± (14.93)	5.30 ± (14.97)	5.83 ± (13.30)	0.561
Pelargonidin	8644	1.70 ± (5.61)	1.72 ± (5.64)	1.31 ± (4.05)	0.916
Peonidin	8644	2.57 ± (15.14)	2.56 ± (15.20)	2.87 ± (12.90)	0.788
Catechin	8644	7.87 ± (9.41)	7.88 ± (9.43)	7.54 ± (8.79)	0.265
Epigallocatechin	8644	16.72 ± (45.68)	16.68 ± (45.72)	18.00 ± (44.33)	0.317
Epicatechin	8644	10.10 ± (14.79)	10.10 ± (14.82)	10.07 ± (13.73)	0.587
Epicatechin 3 gallate	8644	10.69 ± (29.25)	10.66 ± (29.24)	11.79 ± (29.60)	0.907
Epigallocatechin 3 gallate	8644	28.72 ± (94.96)	28.66 ± (95.28)	31.18 ± (83.24)	0.696
Theaflavin	8644	1.56 ± (3.97)	1.55 ± (3.93)	1.80 ± (5.17)	0.984
Thearubigins	8644	88.85 ± (210.57)	88.37 ± (208.51)	105.86 ± (274.21)	0.820
Eriodictyol	8644	0.18 ± (1.15)	0.18 ± (1.16)	0.14 ± (0.88)	0.340
Hesperetin	8644	8.59 ± (18.54)	8.67 ± (18.67)	5.73 ± (12.65)	0.145
Naringenin	8644	3.44 ± (8.51)	3.40 ± (8.33)	4.70 ± (13.31)	0.412
Apigenin	8644	0.23 ± (1.61)	0.23 ± (1.63)	0.21 ± (0.33)	0.818
Luteolin	8644	0.72 ± (1.04)	0.72 ± (1.05)	0.73 ± (0.78)	0.129
Isorhamnetin	8644	0.89 ± (1.69)	0.90 ± (1.70)	0.78 ± (0.91)	0.613
Kaempferol	8644	4.81 ± (6.20)	4.83 ± (6.21)	4.05 ± (5.83)	0.024
Myricetin	8644	1.57 ± (2.40)	1.57 ± (2.40)	1.65 ± (2.58)	0.991
Quercetin	8644	11.31 ± (10.59)	11.30 ± (10.57)	11.54 ± (11.35)	0.555
Theaflavin 3‐3 digallate	8644	1.71 ± (4.39)	1.70 ± (4.35)	1.99 ± (5.71)	0.847
Theaflavin 3q gallate	8644	1.45 ± (3.77)	1.45 ± (3.74)	1.66 ± (4.91)	0.884
Theaflavin 3 gallate	8644	1.23 ± (3.14)	1.22 ± (3.11)	1.44 ± (4.10)	0.701
Gallocatechin	8644	1.68 ± (3.98)	1.68 ± (3.95)	1.82 ± (4.73)	0.138
Total sum of all 29 flavonoids	8644	220.16 ± (388.57)	219.61 ± (386.36)	239.96 ± (460.71)	0.635
Total anthocyanidins	8644	15.61 ± (38.26)	15.58 ± (38.39)	16.47 ± (33.30)	0.580
Subtotal catechins	8644	75.80 ± (192.49)	75.67 ± (192.85)	80.40 ± (179.51)	0.460
Total flavan 3 ols	8644	170.59 ± (370.14)	169.96 ± (367.90)	193.14 ± (442.93)	0.678
Total flavanones	8644	12.21 ± (24.50)	12.26 ± (24.59)	10.57 ± (21.31)	0.439
Total flavones	8644	0.95 ± (2.16)	0.95 ± (2.19)	0.94 ± (0.90)	0.078
Total flavonols	8644	18.58 ± (18.19)	18.60 ± (18.16)	18.02 ± (19.40)	0.488
Total isoflavones	8644	2.22 ± (11.36)	2.26 ± (11.49)	0.82 ± (4.62)	0.291

^a^ *N* not missing (unweighted).

^b^ *n* (survey‐weighted) (%); mean ± (SD).

^c^ Pearson's *X* ^2^: Rao and Scott adjustment; Design‐based Kruskal–Wallis test.

### 3.2 Development and Validation of the Comorbidity Disease Prediction Model

**FIGURE 2:**
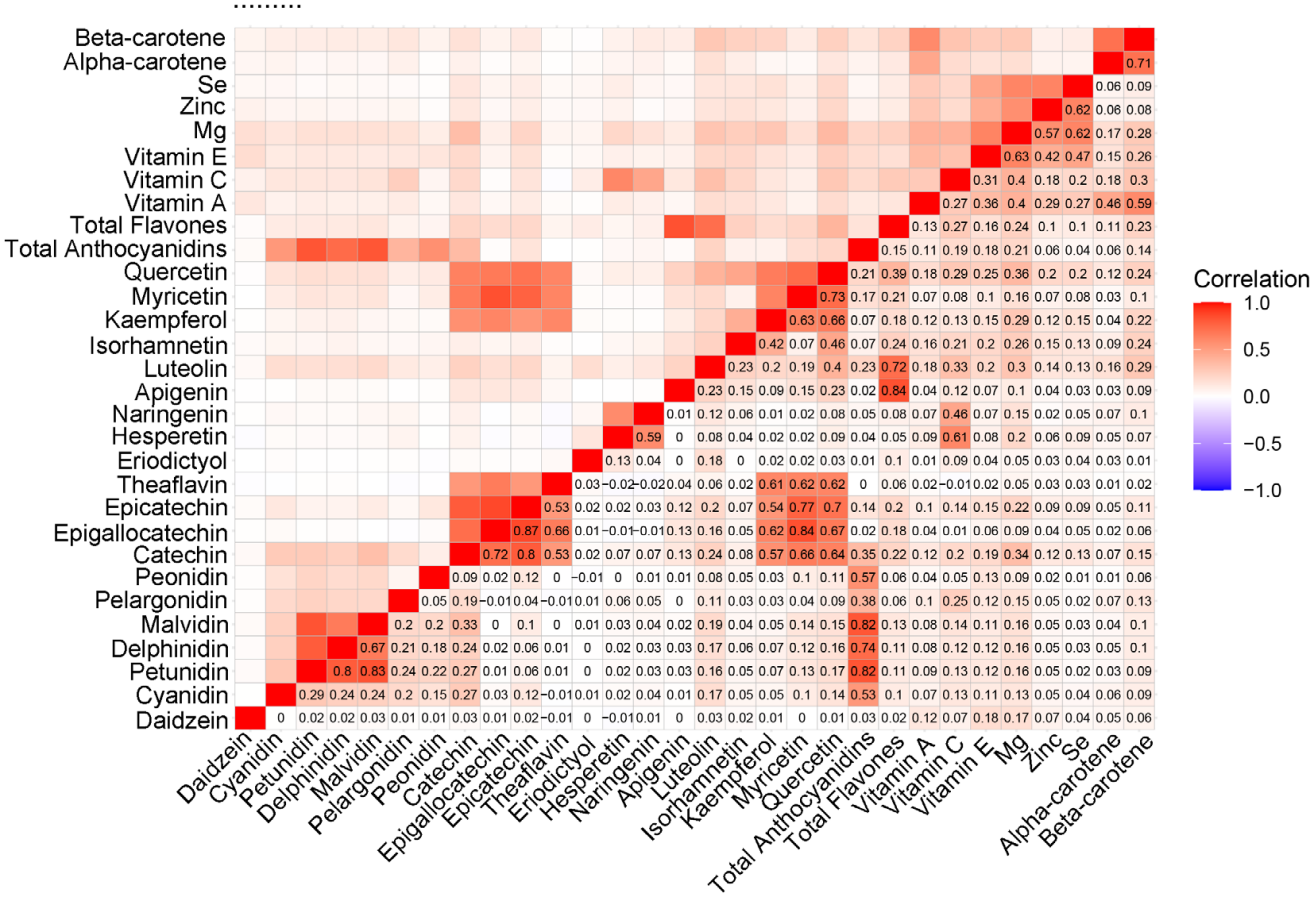
Heatmap of pairwise correlations among dietary antioxidant features after removing those with correlations > 0.9. Strong correlations appear among anthocyanidins (e.g., Cyanidin–Malvidin, *r* = 0.84) and catechins (e.g., Epigallocatechin–Epicatechin, *r* = 0.87).

Before constructing the ML model, we visualized the correlation coefficients between dietary antioxidant features (displayed in Figure S1). From Figure S1, it is evident that some dietary antioxidant features exhibit high correlations, so genistein, glycitein, epicatechin‐3‐gallate, epigallocatechin‐3‐gallate, thearubigins, theaflavin‐3‐3‐digallate, theaflavin‐3q‐gallate, theaflavin‐3‐gallate, gallocatechin, total sum of all 29 flavonoids, subtotal catechins, total flavan‐3‐ols, total flavanones, total flavonols, total isoflavones were removed. Figure 2 illustrates the dietary antioxidant features included in the ML model after addressing collinearity. Finally, the ML model included 30 dietary antioxidant features and 10 baseline features.

**FIGURE 3:**
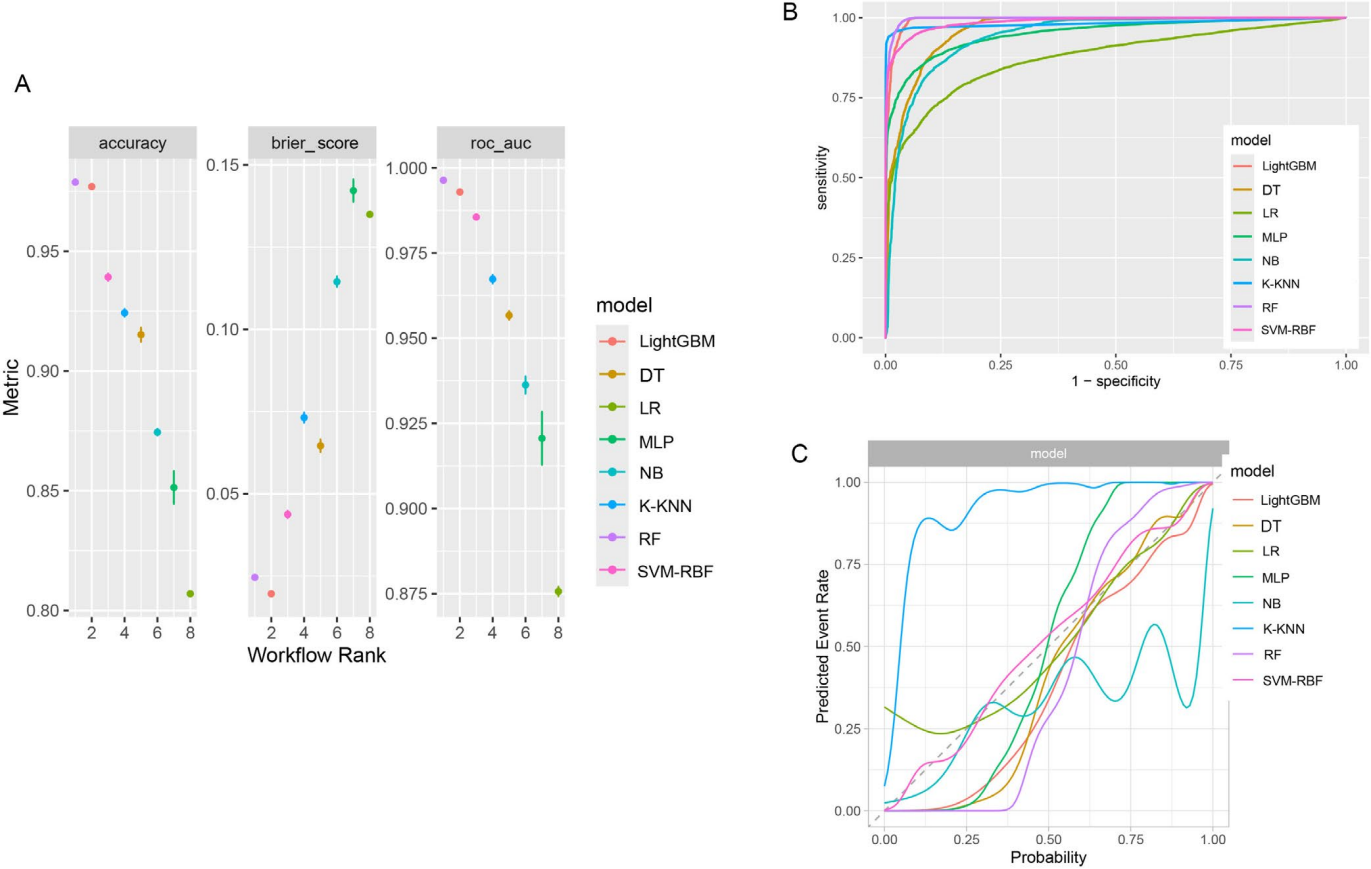
Performance of 8 machine learning models in predicting diabetes and cancer comorbidity. (A) Systematic performance comparison of 8 machine learning algorithms across three evaluation metrics: classification accuracy, Brier score, and area under the ROC curve. (B) Receiver operating characteristic curves. (C) Calibration plots.

Figure 3A presents a systematic performance comparison of eight machine learning algorithms across three evaluation metrics: area under the ROC curve (Figure 3B), classification accuracy, and brier score. The analysis reveals distinct performance stratification among the evaluated models. RF demonstrated superior discriminative capacity with optimal AUC (0.996) and accuracy (0.978) values, suggesting exceptional separation capability. LightGBM closely followed with comparable performance metrics (AUC = 0.993, accuracy = 0.977), indicating its effectiveness as an alternative ensemble method. Regarding probabilistic calibration assessment, both RF (brier score = 0.0241) and LightGBM (brier score = 0.0193) exhibited excellent agreement between predicted probabilities and observed frequencies. Calibration plots (Figure 3C) further support these findings, with the RF curve being nearly aligned with the 45° diagonal between the *X* and *Y* axes, indicating good model consistency. The remaining six models demonstrated comparatively inferior performance across all evaluation dimensions.

### 3.3 Explanation of the Importance of Features Using SHAP Values

**FIGURE 4:**
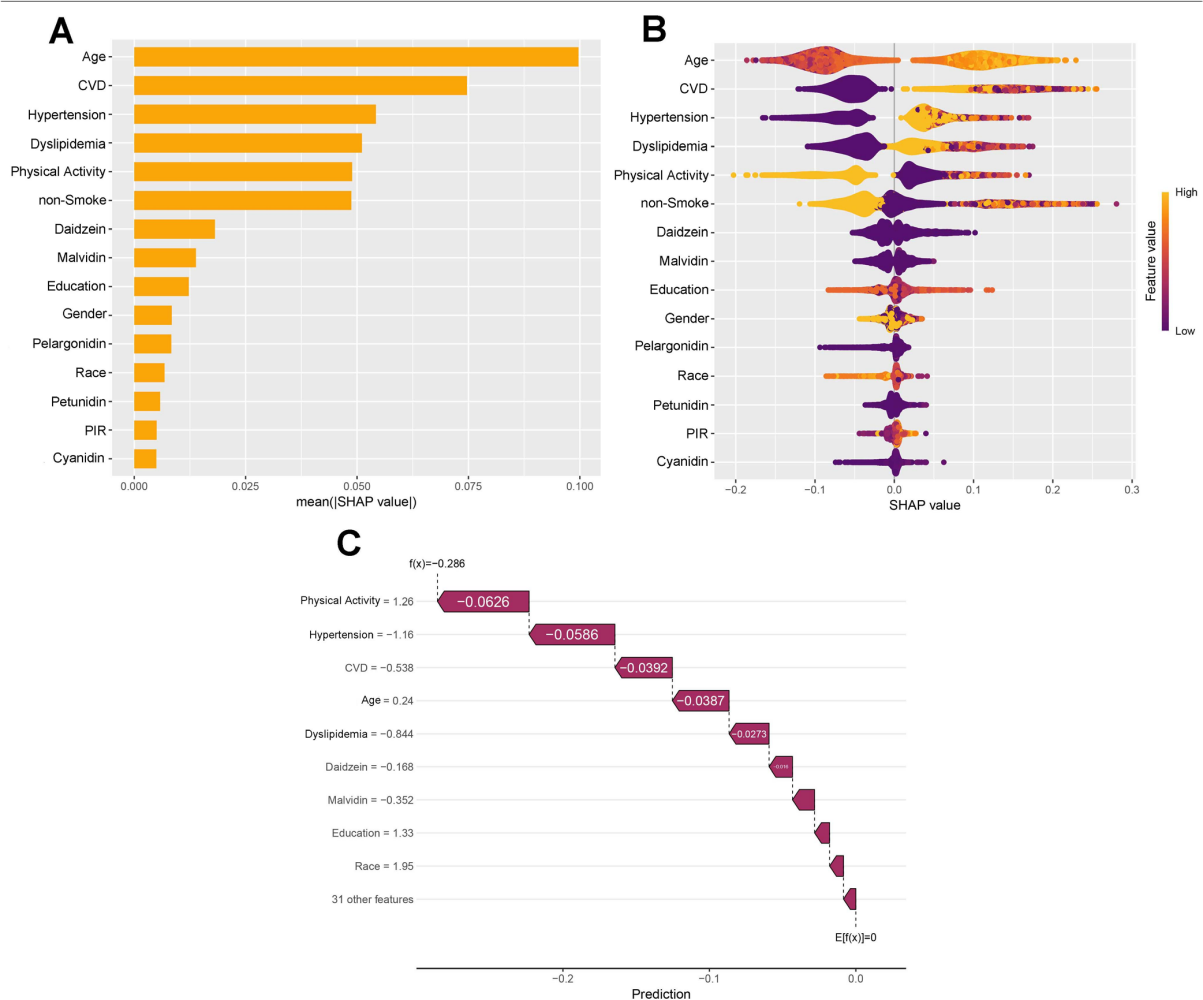
SHAP values of demographic characteristics, clinical baseline features, and dietary antioxidants for RF model. (A) SHAP importance plot. (B) SHAP summary plot. (C) SHAP waterfall plot.

To comprehensively elucidate the predictive mechanisms of the ML model, we integrated demographic characteristics, clinical baseline features, and dietary antioxidants into the SHAP framework. The SHAP importance plot (Figure 4A) displays the top 15 predictors within the optimal RF model. The analysis shows that foundational physiological and cardiometabolic health metrics dominate predictive architecture. Age emerged as the most potent predictor of diabetes‐cancer comorbidity, followed sequentially by CVD, hypertension, dyslipidemia, physical activity, and smoking status. Notably, even when competing against these systemic and behavioral variables, specific dietary antioxidants retained significant predictive value. Daidzein, malvidin, pelargonidin, petunidin, and cyanidin ranked within the top 15 features. As for other dietary antioxidants components, the SHAP importance plot (Figure S2A) illustrates the top 15 dietary antioxidants ranked by importance in predicting comorbidity in the ML model. SHAP values indicate that after daidzein, malvidin, pelargonidin, petunidin, and cyanidin, Mg, delphinidin, epigallocatechin, peonidin, hesperetin, total anthocyanidins, isorhamnetin, kaempferol, eriodictyol, and vitamin C are the primary contributors.

The SHAP summary plot (Figure 4B) further clarifies the directional impact of these features on comorbidity risk. The distribution illustrates that advanced age, alongside positive clinical diagnoses for CVD, hypertension, dyslipidemia, and positive state of smoking, consistently shifts the SHAP values in a positive direction. This identifies them as primary drivers amplifying comorbidity risk. Contrastingly, engagement in moderate to vigorous physical activity and some demographic features such as higher education, female gender, and higher PIR shift the distribution negatively, confirming its substantial protective effect. As indicated by the specific dietary component SHAP summary plot, the intake values of Mg, hesperetin, kaempferol, and vitamin C increase as their corresponding SHAP values decrease, further suggesting their potential protective effects against the comorbidity (Figure S2B).

To illustrate how the model executes individualized clinical predictions, we generated a SHAP waterfall plot using the shapviz package. Figure 4C exhibits the predictive derivation for a specific participant, transitioning from the expected base value (*E*[*f*(*x*)] = 0) to a final localized prediction of −0.286. The plot clearly quantifies the effects of various factors: the patient's active participation in physical activity significantly lowered risk (contribution of −0.0626), while the absence of hypertension (−0.0586), free of CVD (−0.0392), and a younger age (−0.0387) reduced the probability. Operating within the protective framework established by these macro‐level baseline factors, dietary antioxidants such as daidzein and malvidin also provided protective contributions, further reducing the probability of a comorbidity event. This visualization captures the critical impact of baseline health while underscoring the interventional value of antioxidants in personalized prevention. Figure S3C displays the ranking of antioxidants' contributions from a participant in predicting comorbidity events, along with the cumulative prediction level, which ultimately reaches a prediction value of −0.258.

Additionally, we analyzed the relationships between feature values and SHAP values for the top 10 most critical dietary antioxidants via scatter plots (Figure S3). The plots reveal a distinct negative correlation between the intake levels of cyanidin, Mg, delphinidin, and hesperetin and their SHAP values, indicating that increased dietary consumption of these components acts as a protective factor. However, other factors such as daidzein, malvidin, pelargonidin, petunidin, epigallocatechin, and hesperetin did not exhibit a visible linear relationship with SHAP values.

## 4 Discussion

We employed interpretable machine learning methods to explore the relationship between dietary antioxidant intake and the comorbidity of diabetes and cancer using data from the NHANES for the periods 2007–2010 and 2017–2018. Among the eight ML models considered, the RF model exhibited the best performance. The RF model, tested with an AUC of 0.996, demonstrated excellent classification efficiency. SHAP was used to elucidate the importance of each selected feature in the model, identifying key contributors such as daidzein, malvidin, pelargonidin, petunidin, cyanidin, and Mg.

To the best of our knowledge, this is the first study to develop and validate a predictive model for diabetes‐cancer comorbidity, integrating antioxidant factors with baseline characteristics. While our primary focus was on analyzing the contribution of dietary antioxidants, the model also incorporates easily accessible demographic features, lifestyle factors, and personal health conditions, thus enhancing its predictive power. Additionally, we employed the brier score and calibration curves to compare the performance of different models. Machine learning models are increasingly used to explore dietary factors associated with diabetes and cancer. Eckart et al. utilized the XGBoost algorithm to explain the role of diet‐lifestyle patterns in the risk of type 2 diabetes (T2DM), identifying key predictive factors (Eckart and Sharma Ghimire 2025). Xue et al. used decision trees, Random Forest, AdaBoost with decision trees (AdaBoost), and Extreme Gradient Boosting (XGBoost) to identify individuals with T2DM based on physical measurements and dietary questionnaires, concluding that XGBoost performed best (AUC = 0.968) (Xue et al. 2020). Birk et al. explored the use of various machine learning and statistical methods to develop a screening tool for prediabetes using the Global Diet Quality Score (GDQS) and age, with Generalized Linear Mixed Models (GLMM), Generalized Linear Models (GLM), LASSO, and Random Forest techniques showing good performance (AUC > 0.70) (Birk et al. 2021). Park et al. developed a predictive model for sugary carbonated soft drink (CSSB) intake using XGBoost and deep neural network methods, along with dietary factors such as carotenoids, folate, vitamins C and D, calcium, flavonoids, and phenolics, indicating that increased CSSB intake associated with Western‐style diet heightened the risk of metabolic syndrome (Park et al. 2024). In studies linking diet to cancer, dietary factors alone have been shown to create satisfactory predictive models. Abdul et al. employed supervised and unsupervised models using a dietary database to predict colorectal cancer, with the artificial neural network model identified as the best algorithm, achieving a CRC misclassification rate of 1% and a non‐CRC misclassification rate of 3% (Abdul Rahman et al. 2023). Qarmiche et al. applied unsupervised machine learning methods to cluster Moroccan dietary patterns associated with colorectal cancer, based on baseline dietary intake measured by a validated food frequency questionnaire, identifying two cancer‐associated dietary patterns: “risky” and “prudent” (Qarmiche et al. 2023). Yang et al. used Random Forest model to demonstrate that dietary intake of α‐carotene, magnesium, vitamins C and E, lycopene, selenium, lutein, zeaxanthin, and β‐carotene was most beneficial in preventing lung cancer (Yang, Qian, et al. 2023).

In this study, we selected LightGBM, RPART, LR, MLP, NB, K‐KNN, RF, and SVM‐RBF to build predictive models, assessing the features of each model to identify the one most suitable for predicting diabetes‐cancer comorbidity. Our results indicate that the RF model performed best. RF is an ensemble classifier based on majority voting among decision trees, widely used for classification tasks. Increasing evidence suggests that RF demonstrates strong predictive utility in the medical field (Hu et al. 2024; Potash et al. 2020; Zhang et al. 2022). However, another challenge associated with ML models is their clinical interpretability, as these algorithms are often viewed as “black boxes,” lacking clarity on how predictions are made (Wang et al. 2018). To address this, we employed the SHAP method to identify the positive or negative influence of certain features on predictions. Consistent with the “shared soil” hypothesis of chronic systemic diseases, our SHAP analysis identified advancing age, along with CVD, hypertension, and dyslipidemia, as the most significant drivers of diabetes‐cancer comorbidity possibility (Silva et al. 2021). Age serves as an unavoidable proxy for cumulative biological degradation which facilitates oncogenesis and metabolic failure (Petrie et al. 2018; Gao et al. 2024). Concurrently, the predictive weight of CVD and hypertension underscores the severe downstream consequences of endothelial dysfunction and chronic systemic inflammation (Newman et al. 2025; Koene et al. 2016; Katsi et al. 2023). While dyslipidemia was similarly identified as a primary driver of comorbidity risk, the systemic lipotoxicity serves as the incubator for both advanced diabetic organ damage and aggressive oncogenesis (Katsi et al. 2023; Feng et al. 2024). Contrasting with these non‐modifiable risks, our model confirmed moderate vigorous physical activity and specific dietary antioxidants as the most potent modifiable therapeutic targets. Protective SHAP values associated with physical activity reflect its profound physiological capacity to mitigate hyperinsulinemia through insulin‐independent glucose uptake in skeletal muscle while simultaneously downregulating the systemic inflammation that fuels tumor proliferation (Feng et al. 2024; Venkatasamy et al. 2013; Golbidi et al. 2012). The protective SHAP values associated with non‐smoking are consistent with extensive evidence that indicated smoking is a well‐established risk factor for T2DM and multiple malignant neoplasms (Dai et al. 2022). The model also identified female gender, higher education, and higher PIR as protective features, shifting the SHAP distribution negatively. Higher educational attainment is well‐documented to correlate with superior health literacy, enhanced adherence to complex medical and dietary management (Sells Michael et al. 2023). Similarly, the protective effect of female gender may be attributed to the cardiovascular and metabolic protective effects of estrogen during premenopausal years, which dictate a more favorable distribution of adipose tissue and superior baseline insulin sensitivity compared to males (Kautzky‐Willer et al. 2016). Same as epidemiological literature has indicated, a higher PIR (indicating higher levels of poverty) is associated with better health outcomes, a decreased prevalence of metabolic syndrome, and lower cardiovascular mortality (Sells Michael et al. 2023).

As for specific dietary antioxidants, the results highlight that polyphenols (such as cyanidin, hesperetin, kaempferol), minerals (such as magnesium), and vitamins (such as vitamin C) are key predictive factors. Although the dependence plot and summary plot did not show a clear linear relationship, daidzein, malvidin, pelargonidin, petunidin, and peonidin are among the most important features in the model according to the SHAP importance plot. This suggests that they have a significant average effect on the prediction. The inconsistency may indicate that their influence is nonlinear or affected by interaction effects with other features (e.g., daidzein's interaction with kaempferol), leading to greater variability in their SHAP values under similar feature values.

In this study, dietary antioxidant components are primarily categorized into three types: vitamins, minerals, and polyphenols. These small molecules exert their antioxidant effects through various mechanisms. Vitamin A acts as an indirect antioxidant by transcriptionally regulating genes involved in mediating typical antioxidant responses in the human body (Blaner et al. 2021). Vitamin C neutralizes free radicals through electron donation, thereby exerting its antioxidant effects (Getoff 2013). Vitamin E reduces peroxide radicals and forms tocopherol radicals, further modulating the bioactivity and signaling associated with membrane lipids (Niki 2014). Minerals mainly function as cofactors for enzymes involved in oxidative stress; however, their roles extend beyond this. For example, selenium and zinc serve as cofactors for glutathione peroxidase, while magnesium is a cofactor for glutathione peroxidase, superoxide dismutase, and catalase (Morais et al. 2017; Zhang et al. 2023). Carotenoids are thought to possess direct antioxidant activities, primarily functioning as quenchers of singlet oxygen (Böhm et al. 2012). Flavonoids are potent antioxidants in vitro, capable of neutralizing free radicals by donating electrons or hydrogen atoms to various reactive oxygen, nitrogen, and chlorine species, including hydroxyl radicals, peroxide radicals, hypochlorous acid, and peroxynitrite (Panche et al. 2016). Our baseline table reveals unexpected results, showing higher intake of vitamin A and β‐carotene in individuals with comorbidities. This could be due to the potential adverse effects of vitamin A and β‐carotene on cancer (Middha et al. 2019; Omenn et al. 1996). Daidzein and malvidin are two of the most significant antioxidants in this study. While clinical trials have yet to confirm the efficacy of daidzein in the prevention or treatment of diabetes, preclinical studies have demonstrated its benefits, including the modulation of liver enzymes involved in glycolysis, enhancement of glucose uptake in myocytes, reduction in fasting blood glucose, total serum cholesterol, and the homeostasis model assessment index, as well as improvements in HbA1c, insulin, and C‐peptide levels (Cheong et al. 2014; Choi et al. 2008; Sun et al. 2016). Chronic and uncontrolled elevated glucose levels lead to the generation of ROS, which, through the formation of advanced glycation end‐products, activate the polyol, protein kinase C, and hexosamine pathways (Laddha and Kulkarni 2019). These pathways influence hemodynamics, genetics, and metabolism, leading to damage in various organs, including the kidneys, retina, nerves, and heart (Laddha and Kulkarni 2019). Daidzein exhibits both ROS‐scavenging and hypoglycemic properties, effectively preventing the progression of diabetes‐related complications in the kidneys, retina, heart, and bladder (Laddha and Kulkarni 2020, 2021, 2022). Furthermore, daidzein demonstrates broad‐spectrum anticancer activity. Cellular and animal studies have shown that daidzein promotes apoptosis and inhibits proliferation in breast cancer, prostate cancer, colorectal cancer, liver cancer, and neuroblastoma (Guo et al. 2004; Jin et al. 2010; Lo et al. 2007; Park et al. 2013; Szliszka and Krol 2011).

According to SHAP values, malvidin ranks second in importance. Malvidin, one of the most prominent anthocyanins, has a chemical structure similar to delphinidin and is found in various fruits, vegetables, and their derivatives. Malvidin is typically linked to different sugar moieties at the C‐3 position. The molecule contains four hydrogen bond donors, making it a potent ROS scavenger (Husain et al. 2022). Clinical trials have shown that supplementation with anthocyanins (each capsule containing 80 mg of anthocyanins, including 3.0% malvidin‐3‐glucoside, malvidin‐3‐galactoside, and malvidin‐3‐arabinoside) in type 2 diabetes patients significantly reduces fasting blood glucose and the homeostasis model assessment of insulin resistance compared to a placebo group (Li et al. 2015). In patients with prediabetes or newly diagnosed type 2 diabetes, daily supplementation with 320 mg of anthocyanins improved glucose and lipid metabolism (Yang et al. 2021). Type 2 diabetes patients taking a standardized extract from bilberries containing anthocyanins, including malvidin‐3‐galactoside, malvidin‐3‐glucoside, and malvidin‐3‐arabinoside, significantly reduced blood glucose and insulin levels (Hoggard et al. 2013). In vitro and in vivo studies also revealed the anticancer effects of malvidin. Malvidin treatment resulted in a significant reduction in lymphoma volume and increased white blood cell count in mice (Sakthivel et al. 2020). Malvidin‐3‐galactoside modulated pathways related to proliferation, apoptosis, migration, and invasion, showing potential for liver cancer prevention and inducing apoptosis in liver tumor cells in mice (Lin et al. 2020; Wang et al. 2019). Additionally, studies have demonstrated that malvidin induces cell cycle arrest and apoptosis in oral cancer cells via a mitochondrial‐mediated pathway (Baba et al. 2017). Furthermore, malvidin significantly inhibited migration in human glioblastoma cells (Ouanouki et al. 2017).

The integration of interpretable ML into nutritional epidemiology presented in this study yields several practical implications for both clinical practice and broad public health policy. First, by identifying specific dietary antioxidants such as daidzein, malvidin, and magnesium alongside baseline clinical factors that strongly predict the co‐occurrence of diabetes and cancer, healthcare professionals are provided with a multidimensional matrix to better assess patient risk profiles and continuously monitor their health. Second, the granular insights derived from the SHAP analysis transition theoretical population‐level nutrition into actionable, personalized dietary recommendations. By understanding the directional impact and relative importance of specific polyphenols and minerals, clinicians can guide individuals toward antioxidant‐rich diets designed to mitigate the risk of both metabolic and oncological disease progression. Third, the interpretable machine learning models developed, when carefully validated and integrated into electronic health records, could automate complex risk stratification processes and flag high‐risk patients for early preventive screening strategies. Finally, these computational results serve as a data‐driven foundation for future mechanistic and longitudinal research. The pronounced predictive signals associated with compounds like malvidin and daidzein highlight the urgent need for targeted molecular studies to unravel the precise mechanisms by which these antioxidants modulate the insulin and insulin‐like growth factor axis, neutralize reactive oxygen species, and suppress systemic inflammation, thereby contributing to the continuous evolution of evidence‐based lifestyle medicine.

Several limitations of our study must be addressed. First, the NHANES dataset lacks temporal resolution, which prevents causal inferences between antioxidant intake and comorbidity. Second, the reliance on 24‐h dietary recalls without specific day‐of‐week adjustment introduces recall and reporting bias; thus, it may not capture long‐term dietary patterns. Future studies should incorporate food frequency questionnaires or biomarker‐based assessments, such as plasma antioxidant levels, for greater accuracy. External validation in larger, more diverse populations, including multiethnic cohorts, is crucial for ensuring the model's generalizability. Thirdly, our study did not account for factors influencing antioxidant bioavailability, such as the food matrix, cooking methods, and individual metabolism, highlighting the need for future metabolomic analyses. Finally, the cancer status was dependent on self‐reported questionnaire data. While extensive external validation studies indicate that self‐reported cancer possesses exceptionally high specificity and moderate‐to‐high sensitivity, it remains inherently vulnerable to misclassification. Therefore, future prospective longitudinal cohort studies, ideally integrating long‐term dietary tracking technologies, objective metabolomic biomarkers, and registry‐verified oncological clinical data, are urgently warranted to externally validate and refine these predictive computational models.

## 5 Conclusion

In conclusion, we developed predictive models for diabetes‐cancer comorbidity using LightGBM, RPART, LR, MLP, NB, K‐KNN, RF, and SVM‐RBF. Among these 8 algorithms, RF and LightGBM demonstrated superior discriminability and accuracy for predicting diabetes‐cancer comorbidity. SHAP values demonstrated that advanced age, cardiovascular disease, hypertension, and dyslipidemia act as the strongest predictors positively associated with comorbidity risk. We also identified daidzein and malvidin as the primary antioxidants in the model.

## Author Contributions

**Yi Zhang:** conceptualization, methodology, validation, supervision, resources, project administration, writing – review and editing, funding acquisition. **Shuqing Jin:** investigation, visualization, resources, writing – review and editing, writing – original draft, software, data curation. **Yunfeng Liu:** supervision, conceptualization, methodology, validation, formal analysis, investigation, funding acquisition, project administration, resources, writing – review and editing, writing – original draft. **Ming Xu:** conceptualization, methodology, software, data curation, investigation, validation, formal analysis, writing – original draft, writing – review and editing, resources, visualization. **Qinhao Liu:** methodology, investigation, writing – review and editing, resources, validation, software, data curation. **Hongxia Wei:** validation, methodology, investigation, writing – review and editing, visualization. **Yuchen Sun:** investigation, writing – review and editing, formal analysis. **Ru Li:** conceptualization, methodology, investigation, validation, formal analysis, writing – original draft, writing – review and editing, visualization, data curation, software. **Zhe Li:** investigation, validation, writing – review and editing, visualization.

## Funding

This work was supported by the Shanxi Provincial Central Leading Local Science and Technology Development Fund Project (YDZJSX20231A059, YDZJSX2022A059), the Four “Batches” Innovation Project of Invigorating Medical through Science and Technology of Shanxi Province (2023XM022), and the Shanxi Province Higher Education “Billion Project” Science and Technology Guidance Project (BYJL‐024).

## Ethics Statement

Ethics approval for this study was granted by the National Centre for Health Statistics Research Ethics Review Board (Protocol #2005‐06, #2011‐17 and #2018‐01).

## Consent

Since this study involves secondary data analysis, the original informed consent provided during primary data collection included permission for secondary use, eliminating the need for additional participant consent. Participants' privacy was protected by anonymizing or de‐identifying the data to prevent identification. Further details on NHANES ethics approval are available on the CDC's official website: https://www.cdc.gov/nchs/nhanes/about/erb.html?CDC_AAref_Val=https://www.cdc.gov/nchs/nhanes/irba98.htm.

## Conflicts of Interest

The authors declare no conflicts of interest.

## Supporting information


**Figure S1.** The correlation coefficients between dietary antioxidant features.
**Figure S2**. SHAP values of dietary antioxidants for RF model. (A) SHAP importance plot. (B) SHAP summary plot. (C) SHAP waterfall plot.
**Figure S3**. The SHAP values and the correlation scatter plot between top 10 dietary antioxidant features.

## Data Availability

The data that support the findings of this study were derived from the following resources available in the public domain: ‐National Health and Nutrition Examination Survey at https://wwwn.cdc.gov/nchs/nhanes/tutorials/sampledesign.aspx and—FNDDS Flavonoid database at https://www.ars.usda.gov/northeast‐area/beltsville‐md‐bhnrc/beltsville‐human‐nutrition‐research‐center/food‐surveys‐research‐group/docs/fndds‐flavonoid‐database/.
