# Supplementary material for: Machine Learning Unveils Dietary Antioxidants as Influential Factors for Diabetes‐Cancer Comorbidity: Insights From National Health and Nutrition Examination Survey
Source: Food Sci Nutr. 2026 Apr 27;14(5):e71828. doi: 10.1002/fsn3.71828 (PMC13121860; doi:10.1002/fsn3.71828)
Supplement: Supplementary file 1 — Figure S1. The correlation coefficients between dietary antioxidant features. Figure S2. SHAP values of dietary antioxidants for RF model. (A) SHAP importance plot. (B) SHAP summary plot. (C) SHAP waterfall plot. Figure S3. The SHAP values and the correlation scatter plot between top 10 dietary antioxidant features. [file FSN3-14-e71828-s001.docx]

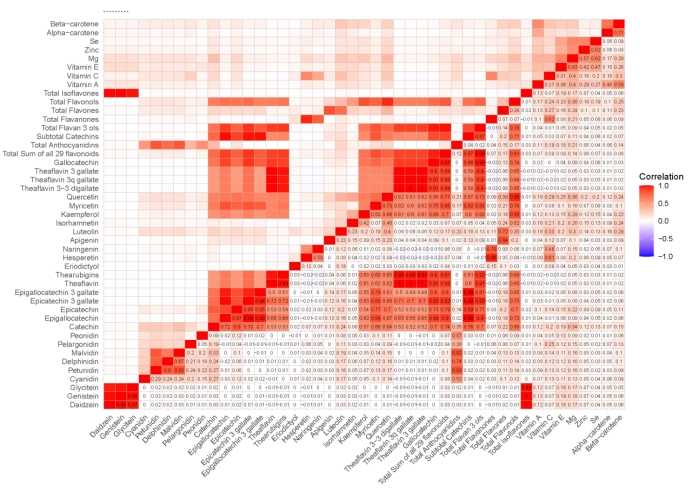


Supplementary Figure 1. The correlation coefficients between dietary antioxidant features.


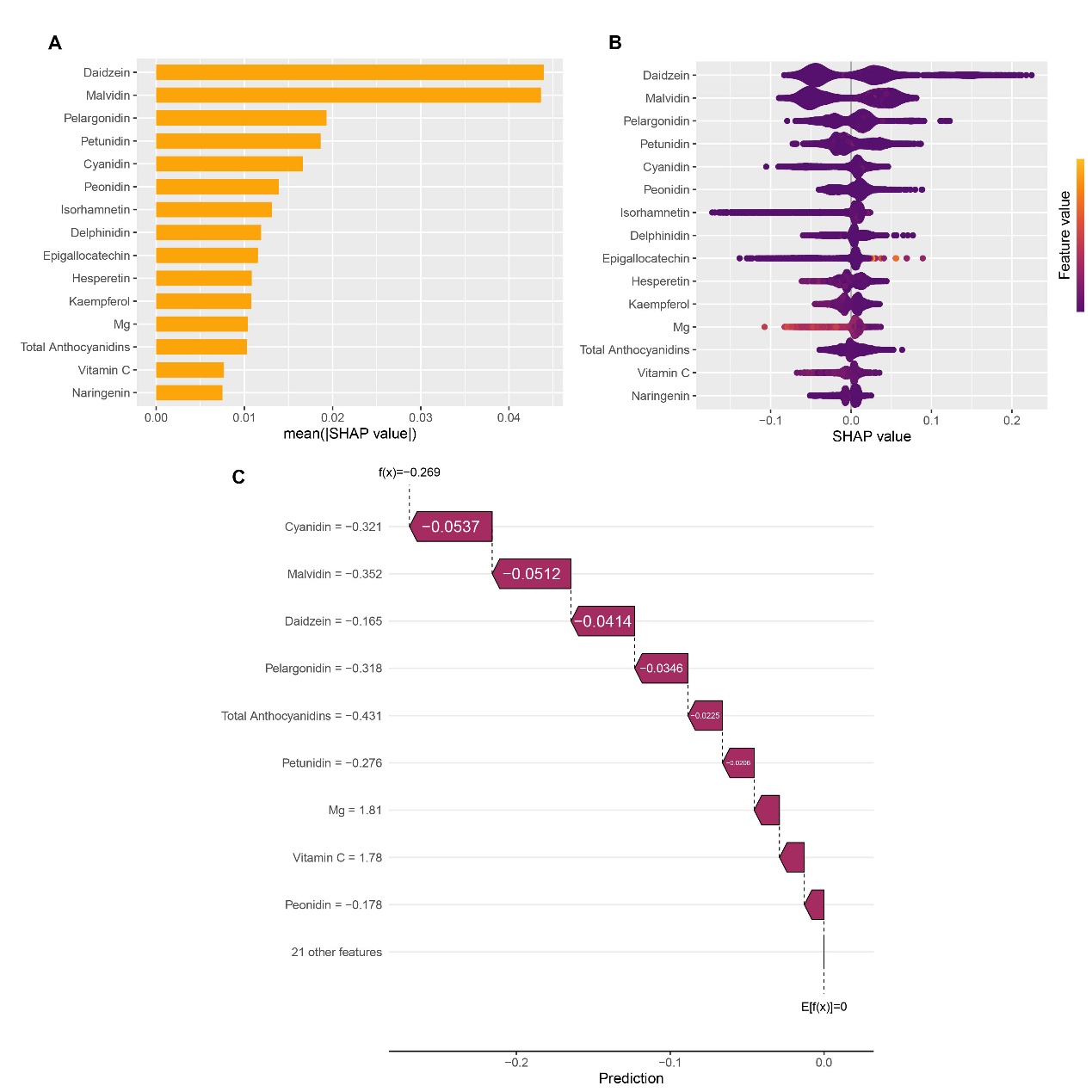


Supplementary Figure 2. SHAP values of dietary antioxidants for RF model. (A) SHAP importance plot. (B) SHAP summary plot. (C) SHAP waterfall plot.


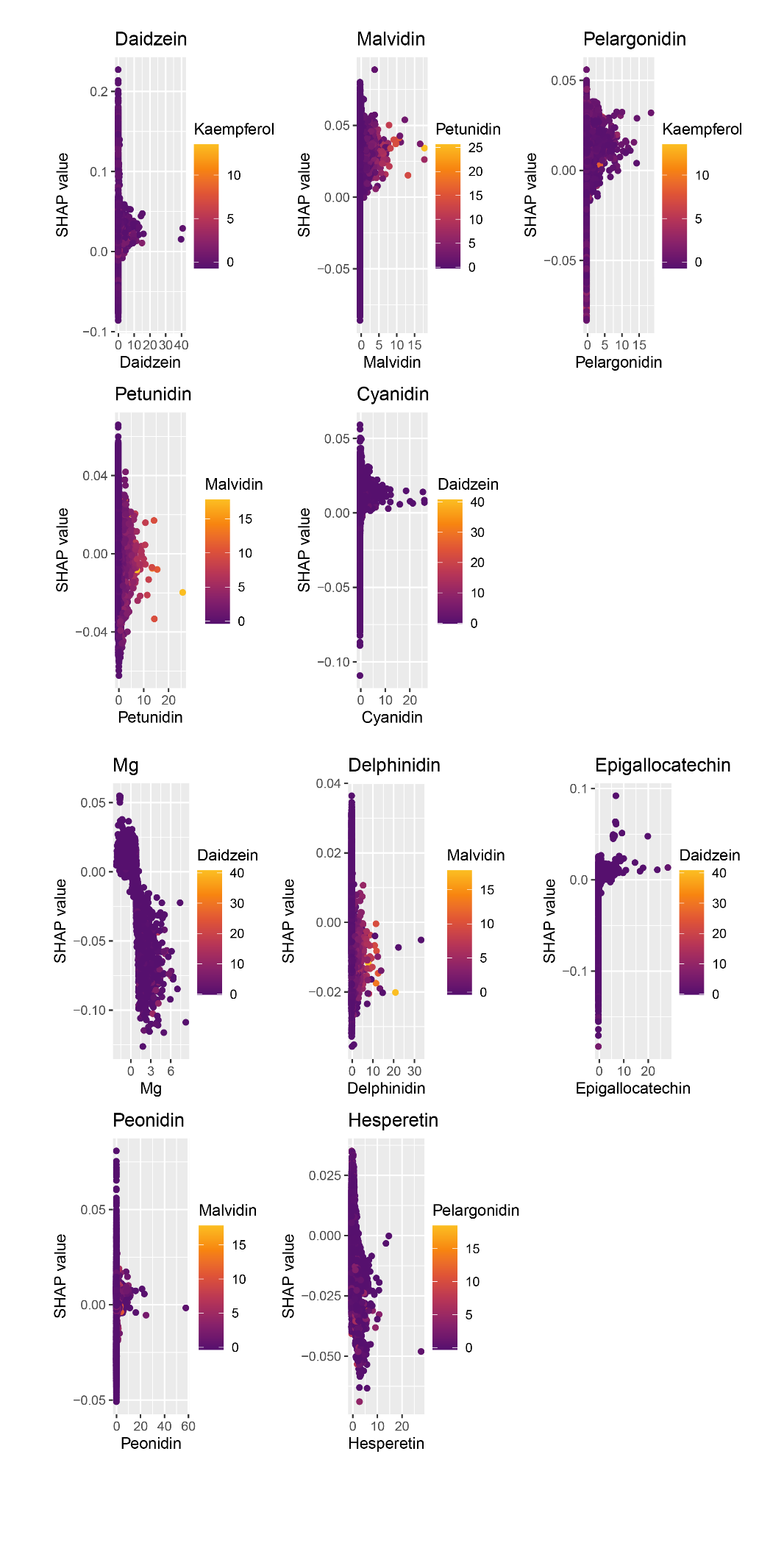


Supplementary Figure 3. The SHAP values and the correlation scatter plot between top 10 dietary antioxidant features.
